# Cell-Free Strategies for Repair and Regeneration of Meniscus Injuries through the Recruitment of Endogenous Stem/Progenitor Cells

**DOI:** 10.1155/2018/5310471

**Published:** 2018-07-12

**Authors:** Weimin Guo, Wenjing Xu, Zhenyong Wang, Mingxue Chen, Chunxiang Hao, Xifu Zheng, Jingxiang Huang, Xiang Sui, Zhiguo Yuan, Yu Zhang, Mingjie Wang, Xu Li, Zehao Wang, Jiang Peng, Aiyuan Wang, Yu Wang, Shuyun Liu, Shibi Lu, Quanyi Guo

**Affiliations:** ^1^Institute of Orthopaedics, Chinese PLA General Hospital, Beijing Key Lab of Regenerative Medicine in Orthopaedics, Key Laboratory of Musculoskeletal Trauma & War Injuries, PLA, No. 28 Fuxing Road, Haidian District, Beijing 100853, China; ^2^First Department of Orthopedics, First Affiliated Hospital of Jiamusi University, No. 348 Dexiang Road, Xiangyang District, Jiamusi 154003, China; ^3^Institute of Anesthesiology, Chinese PLA General Hospital, No. 28 Fuxing Road, Haidian District, Beijing 100853, China; ^4^Department of Orthopedic Surgery, First Affiliated Hospital, Dalian Medical University, No. 222 Zhongshan Road, Xigang District, Dalian 116011, China

## Abstract

The meniscus plays a vital role in protecting the articular cartilage of the knee joint. The inner two-thirds of the meniscus are avascular, and injuries to this region often fail to heal without intervention. The use of tissue engineering and regenerative medicine techniques may offer novel and effective approaches to repairing meniscal injuries. Meniscal tissue engineering and regenerative medicine typically use one of two techniques, cell-based or cell-free. While numerous cell-based strategies have been applied to repair and regenerate meniscal defects, these techniques possess certain limitations including cellular contamination and an increased risk of disease transmission. Cell-free strategies attempt to repair and regenerate the injured tissues by recruiting endogenous stem/progenitor cells. Cell-free strategies avoid several of the disadvantages of cell-based techniques and, therefore, may have a wider clinical application. This review first compares cell-based to cell-free techniques. Next, it summarizes potential sources for endogenous stem/progenitor cells. Finally, it discusses important recruitment factors for meniscal repair and regeneration. In conclusion, cell-free techniques, which focus on the recruitment of endogenous stem and progenitor cells, are growing in efficacy and may play a critical role in the future of meniscal repair and regeneration.

## 1. Introduction

The meniscus is a fibrocartilaginous structure that rests in the joint space between the femoral condyle and tibial plateau cartilage [1] and ensures normal knee joint function [2]. The meniscus is prone to injury, and the incidence of these injuries has been increasing [3]. These types of injuries are challenging to treat, as the inner regions of the meniscus are avascular [4, 5]. If left untreated, injuries in the avascular region will not heal and will inevitably lead to the development of osteoarthritis (OA) [6–8]. The development of tissue engineering and regenerative medicine techniques has provided new hope for the treatment of meniscal defects [9].

Meniscal tissue engineering and regenerative medicine typically use one of two techniques, cell-based or cell-free. In cell-based strategies, repair is done using cellular scaffolds, seed cells, or the application of biochemical and biomechanical stimuli [10]. Cell-based strategies often rely on the expansion of seed cells in vitro, before implantation of the cell-scaffold composite. This step is slow and prone to complications including cell contamination, cell dedifferentiation, and the transmission of disease [11, 12]. Cell-free strategies do not use cell culture, reducing both cost and time to treatment [12]. Therefore, cell-free techniques may have a wider clinical application than cell-based techniques.

Cell-free techniques recruit endogenous stem/progenitor cells to participate in the repair process [13, 14]. Many tissues and organs preserve endogenous stem/progenitor cells throughout their lifespan [15]. After an injury, the local endogenous stem/progenitor cells can be stimulated and recruited to the injured sites, where they gradually restore tissue structure and organ function [16]. Therefore, successful cell-free strategies for meniscus repair and regeneration require application of the appropriate stimulation and recruitment factors [17, 18].

Knowledge of the exact cellular mechanisms for stimulating these endogenous cells is of great importance for tissue repair and regeneration [19]. First, local endogenous stem/progenitor cells must be stimulated in a manner similar to that during tissue injury. These cells must then migrate to the injured site, proliferate, and differentiate. Finally, they must mature and restore tissue function. The critical questions for cell-free strategies are as follows: (1) where are these endogenous cells located and (2) what are the best mechanisms to recruit them? Many studies have been conducted focusing on these two questions. Several have shown that growth factors, chemokines, human serum (HS), and platelet-rich plasma (PRP) may all have a positive effect on cellular migration. Others have found that specific cell markers such as proteoglycan 4 (PRG4) or growth/differentiation factor 5 (GDF-5) play an important role in cartilage repairing and regeneration following knee joint injuries.

This review will summarize existing cell-free techniques for meniscus repair and regeneration, specifically those that recruit endogenous stem/progenitor cells. We first present a systematic analysis and comparison of cell-based and cell-free techniques. Next, we summarize potential sources for endogenous stem and progenitor cells. Finally, we discuss important recruitment factors for meniscal repair and regeneration.

## 2. Cell-Based Strategies for Meniscus Repair and Regeneration

Cell-based strategies include the use of seed cells, cellular scaffolds, and biomechanical or biochemical stimuli. These strategies make up the bulk of classic meniscus tissue engineering techniques. Numerous combinations of seed cells and scaffolds have been used. In the native meniscus, both the cell types and ECM components are heterogeneous and vary by region [20–22]. Cells in the inner region show chondrocyte-like morphology and are surrounded by 60% type II collagen and 40% type I collagen. Cells in the outer region are fibroblast-like and are embedded in an extracellular matrix (ECM) composed of 90% type I collagen. On the surface of the meniscus are fusiform cells that secrete lubricin. Lubricin is chondroprotective and can prevent wear-induced cartilage degradation [23].

Cells taken from the meniscus itself may be the best seed cells for promoting regeneration and repair. Martinek et al. used autologous fibrochondrocytes to seed a collagen-meniscus implant (CMI). The seeded CMI was then implanted into a sheep model of joint injury [24]. Their results showed greater macroscopic and histological improvement in the seeded CMI group when compared to the nonseeded CMI group. Esposito et al. seeded allogeneic fibrochondrocytes into PLDLA/PCL-T (poly(L-co-D,L-lactic acid)/poly(caprolactone-triol)) scaffolds to repair meniscal defects in a rabbit model of joint injury [25]. They showed that these biosynthetic polymer scaffolds restored biomechanical function, could promote fibrocartilaginous tissue formation, and may have prevented articular cartilage degeneration. They also noted that this process was slow and that meniscus regeneration and articular cartilage protection may only occur over long time periods. Finally, Baker et al. showed that human meniscus cells seeded into a scaffold of aligned nanofibers could improve the in vitro biochemical and mechanical properties of tissue-engineered implants [26].

Chondrocytes may also be a viable cell type for use in meniscal tissue engineering. Several studies have shown that chondrocytes possess an excellent capacity to regenerate and have high cartilage-specific ECM expression. As well, chondrocytes may be harvested from the articular cartilage, rather than from the meniscus itself, reducing trauma to the already damaged meniscus in patients with these types of injuries [27]. Kon et al. completely replaced the native meniscus with a tissue-engineered construct consisting of autologous chondrocytes seeded into a hyaluronic acid/polycaprolactone scaffold. They showed that seeding the scaffold with autologous chondrocytes enhanced meniscal regenerative capacity and resulted in improved in vivo fibrocartilaginous tissue formation [28]. Peretti et al. used autologous chondrocytes seeded into devitalized allogenic meniscal slices to regenerate a longitudinal tear in the avascular portion of the meniscus in a pig knee injury model. Histological and histomorphometric analysis showed multiple areas of healing in specimens taken from the experimental group [29]. Jülke et al. demonstrated that expanded autologous chondrocytes in combination with a porcine collagen membrane improved the healing of avascular zone tears when compared to conventional suture repair in a goat knee injury model [30]. Scotti et al. showed that using a chondrocyte-fibrin hydrogel as biologic glue could promote healing between two porcine meniscal slices in a nude mouse model of knee injury [31]. Finally, Forriol et al. showed that the introduction of autologous chondrocytes alongside conventional trephination and suture techniques improved the healing avascular zone tears in an ovine knee injury model [32].

Cell-based meniscal regeneration strategies that use stem cells have also been extensively studied. Of note, mesenchymal stem cells (MSCs) have been intensively investigated since their initial discovery [33–35]. MSCs can be derived from many tissues including bone marrow, adipose, and synovium. Once isolated, MSCs have the unique capacity to differentiate into many mature, terminally differentiated cell types including osteoblasts, chondrocytes, adipocytes, and other types of connective tissue [36]. The specific role of MSCs in tissue regeneration and repair remains controversial [37–40]. Some studies have suggested that MSCs secrete a variety of trophic factors that enhance cellular viability and cellular proliferation and reduce cell apoptosis. It is possible that these factors even modulate immune response to some extent [41]. Several studies have shown that MSCs derived from a variety of connective tissues demonstrated remarkable efficacy in promoting meniscus regeneration and repair (Table 1).

Several additional cell-based strategies including cell coculture, zonal recapitulation, or scaffold-free tissue engineering methods have also been extensively explored [53, 54]. However, many of these studies were conducted in vitro [55]. Further in vivo study is required to investigate the potential clinical application of these techniques to meniscus regeneration and repair.

## 3. Cell-Free Strategies for Meniscus Repair and Regeneration

Cell-free strategies for meniscus repair and regeneration may avoid many of the limitations and pitfalls of cell-based strategies. Therefore, cell-free strategies may have a broader clinical application. There are two kinds of cell-free meniscal scaffolds currently in clinical use: (1) the collagen meniscus implant (CMI; Ivy Sports Medicine, Montvale, NJ) and (2) the Actifit scaffold (Orteq, London, England) [56]. Both have been shown to reduce pain and improve knee function when used to treat partial meniscal defects. The CMI is produced using purified type I collagen taken from bovine Achilles tendons, which is mixed with hyaluronic acid and chondroitin sulfate [57]. In clinical trials, the CMI scaffold was shown to reduce knee pain and enhance knee joint functional scores in appropriately selected patients over a 24-month follow-up period [58]. The Actifit scaffold is made of poly-*ε*-caprolactone acid and polyurethane and has shown favorable biomechanical capacity, due to its slowly absorbed, highly interconnected, and porous structure [59]. Together these characteristics were found to promote cellular migration from the remaining meniscal rim, resulting in the formation of neomenisci. A two-year, prospective case-series study demonstrated that Actifit promoted meniscal regeneration and that the regenerated meniscus prevented OA progression in the knee [60].

Our group and others have shown that biologically derived cell-free scaffolds can promote meniscal regeneration. In a study conducted by our lab, we combined acellular meniscus extracellular matrix (AMECM) and demineralized cancellous bone (DCB) to fabricate a three-dimensional porous AMECM/DCB composite scaffold [61]. This AMECM/DCB composite scaffold showed excellent biomechanical and biocompatibility characteristics. The AMECM/DCB scaffold was then implanted into medial meniscal defects in a rabbit model of knee injury. Outcomes were compared to a total meniscectomy group. Six months after implantation, the AMECM/DCB scaffold group showed meniscal regeneration and the prevention of articular cartilage degeneration. Similarly, Merriam et al. fabricated a biomechanically functional scaffold mimicking the microstructure of the native meniscus [62]. These scaffolds were made of a type I collagen and hyaluronic acid sponge and reinforced with a tyrosine-derived, biodegradable polymer. Previous studies had shown that these scaffolds could convert a portion of the axial compressive load produced at the knee by body weight into circumferential tensile loads in a manner similar to that of the native meniscus. Implantation into ovine models of knee injury demonstrated that these novel fiber-reinforced meniscal scaffolds could act as functional meniscal substitutions and protect the articular cartilage from further degeneration following total meniscectomy. Recently, a 52-week study has reinforced the conclusion that these meniscal scaffolds could successfully regenerate the meniscus and protect articular cartilage from damage [63].

A variety of growth factors and small proteins may also contribute to meniscal regeneration. Lee et al. used spatially released human growth factors loaded onto the surface of a three dimensionally (3D) printed PCL meniscus scaffold to regenerate a functional, heterogeneous meniscus in a sheep model [64]. Human connective tissue growth factor (CTGF) and transforming growth factor-*β*3 (TGF*β*3) were differentially coated onto the inner and outer zones of the PCL scaffold. Following implantation of the scaffold, the resulting regenerated meniscus displayed zone-specific ECM properties, with newly formed type I collagen in the outer zone and newly formed type II collagen in the inner zone. This distribution was similar to that of the native meniscus. The authors speculated that meniscal regeneration may be driven by the remaining native meniscus, as well as synovium stem/progenitor cells. Pan et al. demonstrated that the conditional deletion of the EGFR gene increased partial meniscectomy-induced ECM secretion in a mouse model. This increase was equivalent to that seen when using the EGFR inhibitor gefitinib [65]. They combined intra-articular delivery of gefitinib with the implantation of a collagen scaffold to repair meniscal defects in a rabbit meniscectomy model. Their results showed that this promoted both meniscal regeneration and prevented OA development.

Platelet-rich fibrin (PRF) is also being used in clinical practice [66–68]. Wong et al. showed that PRF enhanced cellular migration and promoted both meniscocyte proliferation and meniscocyte ECM secretion in vitro [69]. PRF has also been proven to promote meniscal repair in a rabbit meniscal defect model. These studies show that cell-free techniques using PRF represent a novel approach to meniscal regeneration and repair.

## 4. Endogenous Stem/Progenitor Cells Involved in Meniscus Repair and Regeneration

The use of endogenous stem/progenitor cells in meniscal regeneration and repair remains controversial. Still, several recent studies have shown that endogenous stem/progenitor cells derived from the outside of the meniscus or synovium may be responsible for healing following injury.

Meniscal tears in the vascular region typically heal much better than those in the avascular region [70]. Osawa et al. attempted to explore the potential mechanism of this healing. They hypothesized that the vascular region may possess a richer supply of vascular-derived stem cells. Their work showed that in both adult humans and human fetuses, the avascular region had fewer cells expressing CD34 and CD146 than the outer vascular region [71]. Meniscal cells positive for CD34 and CD146 displayed the potential for multilineage differentiation and were more robust than cells isolated from the avascular region. Finally, fetal CD34+ and CD146+ cells, when injected into the knee joints of an athymic rat meniscal tear model, mobilized into the injury site and promoted meniscal repair. Therefore, perivascular stem cells derived from peripheral meniscus may play a role in the endogenous regeneration and repair process following meniscal injury.

Seol et al. demonstrated that endogenous meniscus progenitor cells (MPCs) could be found within meniscal defects and that these cells possessed progenitor-like characteristics [72]. MPCs exhibited low expression of cartilage ECM components (CHAD, COL2A1, COL10A1, and COMP) and high expression of progenitor cell markers (CD44 and Notch1). Overall, the genes expressed by MPCs were similar to those expressed by chondrogenic progenitor cells (CPCs). Of note, MPCs expressed slightly higher levels of the proinflammatory IL and CXCL genes and had higher expression of the protease gene MMP, then CPCs. These results indicated that MPCs may promote inflammation and influence immune cell migration. Moreover, the CXCL and MMP genes also promote endothelial and hematopoietic cell migration. The expression of CXCL12, the gene encoding stromal cell-derived factor 1 (SDF-1), was also upregulated after meniscectomy and was shown to be involved in cell homing following the intra-articular injection of human MPCs. It is possible that meniscal injuries may not heal spontaneously under normal circumstances but that healing may be induced through the application of chemotactic agents and growth factors that regulate MPC migration and differentiation.

Mesenchymal stem/progenitor cells (MSCs) in the synovial membrane and synovial fluid have the potential to differentiate into cartilage; however, whether these cells contribute to cartilage or meniscus regeneration in vivo is still a matter of debate. Kurth et al. used iododeoxyuridine (IdU) and chlorodeoxyuridine (CIdU) in a double nucleoside analog cell-labeling scheme, to identify the role of endogenous synovium-derived stem cells in the in vivo repair of injuries to the articular cartilage [73]. Their results showed that MSCs resident in the knee joint synovium proliferate and undergo chondrogenic differentiation after cartilage injury. In another study, Decker et al. found that knee joint progenitor cells could produce nonmigratory progeny and form distinct local tissues in both the pre- and postnatal period, in a novel GDF5CreERT2 (GDF5-CE), PRG4-CE, and Dkk3-CE mouse model [74]. Progenitor cell tracing at juvenile stages has shown that injury to the articular cartilage can induce a massive and rapid increase in PRG4+ and CD44+/P75+ cells within the synovium and that these cells will later fill the site of injury. These results provide evidence that synovial PRG4+ progenitors may be exquisitely responsive to cartilaginous injury in the acute phase and may be at the forefront of joint tissue regeneration and repair.

During embryonic development, mesenchymal tissue forms the joint interzone (JI). This tissue eventually contributes to the formation of the cartilage template that guides limb development [75, 76]. This mesenchymal tissue is characterized by the expression of a number of genes, including growth and differentiation factor 5 (GDF5). Roelofs et al. explored the role of GDF5 in joint development and cartilage injury repair [77]. Using lineage tracing in a GDF5-Cre mouse model, they showed that GDF5-expressing interzone cells participated in synovial hyperplasia, were able to migrate to perivascular regions with a high expression of Nestin-GFP, and contributed to cartilage repair. Moreover, they characterized the cofactor Yap as a critical transcription factor and showed that it was highly expressed following cartilaginous injury. The conditional silencing of Yap in GDF5-lineage cells inhibited synovial hyperplasia and reduced the overall contribution of GDF5-lineage cells to cartilage regeneration. Together, these results suggest that GDF5-expressing cells may initiate and potentiate endogenous cartilage regeneration and repair.

Mak et al. showed that the intra-articular injection of Sca-1+ GFP+ synovial cells into a C57BL6 mouse model of cartilage injury led to cartilage repair after four weeks [78]. However, GFP expression was only observed at the injury site two weeks after the initial insult and was completely absent at four weeks. This study showed that endogenous stem/progenitor cells derived from synovium, regardless of strain background, were beneficial to cartilage regeneration and repair.

## 5. Recruitment Factors for Endogenous Cell Homing in Meniscal Repair and Regeneration

A number of recruitment factors are important for endogenous cell homing in meniscal regeneration and repair and include growth factors, chemokines, PRP, and ECM. A wide variety of growth factor-based strategies have been applied to meniscal regeneration that take advantage of this property. Work by Lee et al. showed that CTGF and TGF-*β*3, spatially released from a 3D-printed scaffold, could recruit endogenous stem/progenitor cells and promote meniscal regeneration in a sheep model of knee injury [64]. PDGF-AB has also been demonstrated to have a robust effect on cell migration. Qu et al. demonstrated that sequentially releasing active collagenase followed by PDGF-AB could attract local meniscal cells and promote injury repair [79]. The effectiveness of pretreating with collagenase suggests that ECM porosity plays a significant role in cell migration, particularly through connective tissue. Bhargava et al. have shown that both PDGF and HGF have a chemotactic effect on meniscal cells in vitro and that combined PDGF-HGF may further promote the repair of meniscal injuries [80]. Similarly, endothelin-1 (ET-1) and stromal cell-derived factor-1 (SDF-1) have also been shown to stimulate cell migration and enhance meniscal regeneration and repair [81, 82].

Fibroblast growth factor 2 (FGF-2) stabilized by incorporation into gelatin hydrogels (GHs) had shown the ability to enhance the healing of meniscal tear in rabbit model [83]. These biodegradable gelatin hydrogels incorporating FGF-2, on the one hand, may recruit endogenous stem cells to the meniscus tear; on the other hand, GHs incorporating FGF-2 can strongly enhance proliferation and inhibit the death of meniscal cells. Ozeki et al. had shown that transplantation of the Achilles tendon treated with BMP-7 displayed a better meniscal regeneration and articular cartilage protection effect than those in the tendon transplantation alone group in a rat model [84]. The addition of BMP-7 could enhance the fibrocartilage differentiation of tendon cells and meniscus-specific matrix biosynthesis. They convinced that the regenerated meniscus was derived from both donor and host cells by using LacZ-transgenic rat-tracing approach. There is no doubt that the endogenous cells had involved in the meniscus regeneration process. Furthermore, Forriol et al. had attempted to use the BMP-7 mixed with a cellulose putty carrier (OP-1 Putty®) to repair meniscus defects in the avascular area in sheep model. The results had demonstrated some migrated cells present inside the defects and nothing in the control groups after 12 weeks [85]. One year later, their groups also showed that BMP-7 associated with trephination and suture approaches could enhance the healing process of the longitudinal tears in the avascular meniscus in sheep model [32]. To note, Zhang et al. had displayed that local administration of simvastatin could activate the regeneration of an avascular meniscus in the rabbit model [86]. They just speculate that the simvastatin may directly recruit the endogenous stem cells or by the upregulation of BMPs to regenerate the meniscus defect.

In contrast, some inflammatory factors may inhibit cell homing. McNulty et al. demonstrated that pathophysiologic concentrations of both IL-1 and TNF*α* significantly reduce cell migration and tissue formation at the meniscus interface [87]. The addition of IL-1Ra or TNF mAb to explants could potentially prevent the adverse effects of IL-1 or TNF*α*, respectively, and may constitute a future strategy to promote repair following meniscal injury.

Blood-derived products are a promising source for autologous biochemical stimuli that may promote cellular recruitment, proliferation, and differentiation. Several blood-derived products including human serum (HS) and various platelet concentrates have been extensively studied both in vitro and in vivo, to determine their effects on meniscal regeneration. Freymann et al. showed that solutions of 10% HS, 5% conditioned plasma (ACP), and 5% PRP all robustly attracted human meniscus cells [88]. PRP is produced by collecting platelet suspensions from plasma and has a higher platelet concentration than blood [89]. The release of alpha-granules from activated platelets may play a role in the tissue regeneration process [90]. ACP was designed for direct application in clinic and is characterized by a high platelet and low leukocyte concentration when compared to PRP. In one study, the authors explored the effects of HS and PRP on human meniscus cells isolated from patients with early or advanced cartilage degeneration. They showed that the application of 2.5%–30% PRP or 10% HS resulted in meniscal cell recruitment in both groups [91]. Similarly, Wong et al. showed that platelet-rich fibrin (PRF) could promote cellular migration and enhance both the proliferation and ECM synthesis of meniscocytes [69]. PRF is an autogenous fibrin-based biomaterial. It avoids the primary disadvantage of PRP, namely, that PRP typically given by injection and even implanted with scaffolds may result in unexpected risk or lack integration.

Physical stimulation may also play a role in the recruitment of endogenous cells and the promotion of meniscal regeneration and repair. It is well known that the application of electrical stimuli to the articular cartilage can promote repair [92–94]. Similarly, Yuan et al. showed that electrical stimulation could directly induce meniscus cell migration and increase connective tissue strength [95].

Finally, biological scaffolds derived from ECM are widely used for meniscal regeneration and repair. Reing et al. show that the degradation products of ECM scaffolds could be modulators of the recruitment and proliferation of cell types involved in the remodeling process [96].

## 6. Future Prospects for Meniscus Regeneration and Repairing

There has been considerable innovation with regard to cell-free techniques to promote repair and regeneration following meniscal injury. Increasingly, these techniques have included cell-free scaffolds. Nevertheless, it is important to explore the growth factors that mobilize endogenous stem/progenitor cells and promote repair. The spatial release of multiple growth factors from scaffolds represents a promising future avenue for scaffold design.

**Figure 1:**
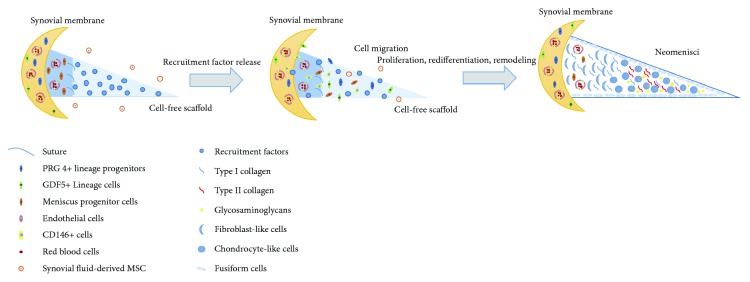
The potentially regenerative mechanism of cell-free strategies for repair and regeneration of meniscus injuries through the recruitment of endogenous stem/progenitor cells.

Recent studies have focused primarily on how to recruit endogenous cells. Still, when the endogenous stem/progenitor cells are at the site of injury, additional factors are required to induce the cellular proliferation, differentiation, and maturation that ultimately results in the regeneration of functional tissue. The potentially regenerative mechanism may be illustrated as Figure 1 based on the previous studies. It is therefore necessary to fully uncover the specific endogenous stem/progenitor cells that best promote meniscus repair and regeneration. The use of genetically modified or gene knockout animal models may aid in the study of both the source of endogenous cells and the specific cell markers that promote injury repair. The identification of these cells and markers will allow for more accurate cell-free scaffolds to be designed. Finally, the advent of 3D printing may allow for the construction of meniscal scaffolds that are appropriately heterogeneous and patient specific.

## 7. Conclusion

Injuries to the avascular region of the meniscus pose a significant clinical challenge. However, cell-free strategies for recruiting the endogenous stem/progenitor cells that promote meniscal repair are a promising avenue for treating these types of injuries. As the mechanisms underlying repair are better understood, more effective cell-free scaffolds will be produced with the goal of eventually achieving a full functional regeneration of the meniscus.

## Figures and Tables

**Table 1:** Stem cell-based strategies for meniscus regeneration.

Animal model	Cell source	Observation time	Authors
Rats	Human BMSCs	8 weeks	Yuan et al. [42]
Rabbits	Autologous BMSCs	24 weeks	Zhang et al. [43]
Rats	Allogeneic BMSCs	8 weeks	Qi et al. [44]
Rabbits	Allogeneic ADSCs	7 months	Moradi et al. [45]
Rabbits	Allogeneic ADSCs	12 weeks	Toratani et al. [46]
Rabbits	ADSCs	12 weeks	Qi et al. [47]
Pigs	Allogeneic SMSs	16 weeks	Hatsushika et al. [48]
Pigs	Allogeneic SMSs	12 weeks	Nakagawa et al. [49]
Rats	Allogeneic SMSs	8 weeks	Ozeki et al. [50]
Rabbits	IPFP	8 weeks	Oda et al. [51]
Rabbits	Human T-MSCs	10 weeks	Koh et al. [52]

BMSC: bone marrow-derived mesenchymal stem cells; ADSCs: adipose-derived mesenchymal stem cells; SMSs: synovium-derived mesenchymal stem cells; IPFP: infrapatellar fat pad; T-MSCs: tonsil-derived mesenchymal stem cells.

## References

[1] Frizziero A., Ferrari R., Giannotti E., Ferroni C., Poli P., Masiero S. (2012). The meniscus tear: state of the art of rehabilitation protocols related to surgical procedures. *Muscles, Ligaments and Tendons Journal*.

[2] Makris E. A., Hadidi P., Athanasiou K. A. (2011). The knee meniscus: structure-function, pathophysiology, current repair techniques, and prospects for regeneration. *Biomaterials*.

[3] Abrams G. D., Frank R. M., Gupta A. K., Harris J. D., McCormick F. M., Cole B. J. (2013). Trends in meniscus repair and meniscectomy in the United States, 2005-2011. *The American Journal of Sports Medicine*.

[4] Cooper D. E., Arnoczky S. P., Warren R. F. (1991). Meniscal repair. *Clinics in Sports Medicine*.

[5] Longo U. G., Campi S., Romeo G., Spiezia F., Maffulli N., Denaro V. (2012). Biological strategies to enhance healing of the avascular area of the meniscus. *Stem Cells International*.

[6] Lee B. S., Bin S. I., Kim J. M. (2016). Articular cartilage degenerates after subtotal/total lateral meniscectomy but radiographic arthrosis progression is reduced after meniscal transplantation. *The American Journal of Sports Medicine*.

[7] Englund M., Paradowski P. T., Lohmander L. S. (2004). Association of radiographic hand osteoarthritis with radiographic knee osteoarthritis after meniscectomy. *Arthritis and Rheumatism*.

[8] Englund M., Guermazi A., Roemer F. W. (2009). Meniscal tear in knees without surgery and the development of radiographic osteoarthritis among middle-aged and elderly persons: the multicenter osteoarthritis study. *Arthritis and Rheumatism*.

[9] Scotti C., Hirschmann M. T., Antinolfi P., Martin I., Peretti G. M. (2013). Meniscus repair and regeneration: review on current methods and research potential. *European Cells and Materials*.

[10] Haddad B., Haddad B., Konan S., Adesida A., Khan W. S. (2013). A systematic review of tissue engineered meniscus and replacement strategies: preclinical models. *Current Stem Cell Research & Therapy*.

[11] Tan G.-K., Dinnes D. L. M., Butler L. N., Cooper-White J. J. (2010). Interactions between meniscal cells and a self assembled biomimetic surface composed of hyaluronic acid, chitosan and meniscal extracellular matrix molecules. *Biomaterials*.

[12] Demirbag B., Huri P. Y., Kose G. T., Buyuksungur A., Hasirci V. (2011). Advanced cell therapies with and without scaffolds. *Biotechnology Journal*.

[13] Ko I. K., Lee S. J., Atala A., Yoo J. J. (2013). *In situ* tissue regeneration through host stem cell recruitment. *Experimental & Molecular Medicine*.

[14] Chen F. M., Wu L. A., Zhang M., Zhang R., Sun H. H. (2011). Homing of endogenous stem/progenitor cells for *in situ* tissue regeneration: promises, strategies, and translational perspectives. *Biomaterials*.

[15] Klimczak A., Kozlowska U. (2016). Mesenchymal stromal cells and tissue-specific progenitor cells: their role in tissue homeostasis. *Stem Cells International*.

[16] Yang C., Jiang J., Yang X., Wang H., Du J. (2016). Stem/progenitor cells in endogenous repairing responses: new toolbox for the treatment of acute lung injury. *Journal of Translational Medicine*.

[17] Ko I. K., Ju Y. M., Chen T., Atala A., Yoo J. J., Lee S. J. (2012). Combined systemic and local delivery of stem cell inducing/recruiting factors for *in situ* tissue regeneration. *The FASEB Journal*.

[18] Ju Y. M., Atala A., Yoo J. J., Lee S. J. (2014). In situ regeneration of skeletal muscle tissue through host cell recruitment. *Acta Biomaterialia*.

[19] Rennert R. C., Sorkin M., Garg R. K., Gurtner G. C. (2012). Stem cell recruitment after injury: lessons for regenerative medicine. *Regenerative Medicine*.

[20] Chevrier A., Nelea M., Hurtig M. B., Hoemann C. D., Buschmann M. D. (2009). Meniscus structure in human, sheep, and rabbit for animal models of meniscus repair. *Journal of Orthopaedic Research*.

[21] Nakata K., Shino K., Hamada M. (2001). Human meniscus cell: characterization of the primary culture and use for tissue engineering. *Clinical Orthopaedics and Related Research*.

[22] Athanasiou K. A., Responte D. J., Brown W. E., Hu J. C. (2015). Harnessing biomechanics to develop cartilage regeneration strategies. *Journal of Biomechanical Engineering*.

[23] Musumeci G., Loreto C., Carnazza M. L., Cardile V., Leonardi R. (2013). Acute injury affects lubricin expression in knee menisci: an immunohistochemical study. *Annals of Anatomy - Anatomischer Anzeiger*.

[24] Martinek V., Ueblacker P., Bräun K. (2006). Second generation of meniscus transplantation: in-vivo study with tissue engineered meniscus replacement. *Archives of Orthopaedic and Trauma Surgery*.

[25] Esposito A. R., Moda M., Cattani S. M. M. (2013). PLDLA/PCL-T scaffold for meniscus tissue engineering. *BioResearch Open Access*.

[26] Baker B. M., Nathan A. S., Huffman G. R., Mauck R. L. (2009). Tissue engineering with meniscus cells derived from surgical debris. *Osteoarthritis and Cartilage*.

[27] Marsano A., Millward-Sadler S. J., Salter D. M. (2007). Differential cartilaginous tissue formation by human synovial membrane, fat pad, meniscus cells and articular chondrocytes. *Osteoarthritis and Cartilage*.

[28] Kon E., Filardo G., Tschon M. (2012). Tissue engineering for total meniscal substitution: animal study in sheep model—results at 12 months. *Tissue Engineering Part A*.

[29] Peretti G. M., Gill T. J., Xu J. W., Randolph M. A., Morse K. R., Zaleske D. J. (2004). Cell-based therapy for meniscal repair: a large animal study. *The American Journal of Sports Medicine*.

[30] Jülke H., Mainil-Varlet P., Jakob R. P., Brehm W., Schäfer B., Nesic D. (2015). The role of cells in meniscal guided tissue regeneration: a proof of concept study in a goat model. *Cartilage*.

[31] Scotti C., Pozzi A., Mangiavini L. (2009). Healing of meniscal tissue by cellular fibrin glue: an in vivo study. *Knee Surgery, Sports Traumatology, Arthroscopy*.

[32] Forriol F., Longo U. G., Duart J. (2015). VEGF, BMP-7, Matrigel™, hyaluronic acid, in vitro cultured chondrocytes and trephination for healing of the avascular portion of the meniscus. An experimental study in sheep. *Current Stem Cell Research & Therapy*.

[33] Friedenstein A. J., Ivanov-Smolenski A. A., Chajlakjan R. K. (1978). Origin of bone marrow stromal mechanocytes in radiochimeras and heterotopic transplants. *Experimental Hematology*.

[34] Friedenstein A. J. (1976). Precursor cells of mechanocytes. *International Review of Cytology*.

[35] Friedenstein A. J., Piatetzky-Shapiro I. I., Petrakova K. V. (1966). Osteogenesis in transplants of bone marrow cells. *Journal of Embryology and Experimental Morphology*.

[36] Caplan A. I. (2007). Adult mesenchymal stem cells for tissue engineering versus regenerative medicine. *Journal of Cellular Physiology*.

[37] Linero I., Chaparro O. (2014). Paracrine effect of mesenchymal stem cells derived from human adipose tissue in bone regeneration. *PLoS One*.

[38] Saeed H., Ahsan M., Saleem Z. (2016). Mesenchymal stem cells (MSCs) as skeletal therapeutics - an update. *Journal of Biomedical Science*.

[39] Korpershoek J. V., de Windt T. S., Hagmeijer M. H., Vonk L. A., Saris D. B. F. (2017). Cell-based meniscus repair and regeneration: at the brink of clinical translation? A systematic review of preclinical studies. *Orthopaedic Journal of Sports Medicine*.

[40] Wang X., Wang Y., Gou W., Lu Q., Peng J., Lu S. (2013). Role of mesenchymal stem cells in bone regeneration and fracture repair: a review. *International Orthopaedics*.

[41] Spees J. L., Lee R. H., Gregory C. A. (2016). Mechanisms of mesenchymal stem/stromal cell function. *Stem Cell Research & Therapy*.

[42] Yuan X., Wei Y., Villasante A. (2017). Stem cell delivery in tissue-specific hydrogel enabled meniscal repair in an orthotopic rat model. *Biomaterials*.

[43] Zhang Z. Z., Wang S. J., Zhang J. Y. (2017). 3D-printed poly(*ε*-caprolactone) scaffold augmented with mesenchymal stem cells for total meniscal substitution: a 12- and 24-week animal study in a rabbit model. *The American Journal of Sports Medicine*.

[44] Qi Y., Chen G., Feng G. (2016). Osteoarthritis prevention and meniscus regeneration induced by transplantation of mesenchymal stem cell sheet in a rat meniscal defect model. *Experimental and Therapeutic Medicine*.

[45] Moradi L., Vasei M., Dehghan M. M., Majidi M., Farzad Mohajeri S., Bonakdar S. (2017). Regeneration of meniscus tissue using adipose mesenchymal stem cells-chondrocytes co-culture on a hybrid scaffold: in vivo study. *Biomaterials*.

[46] Toratani T., Nakase J., Numata H. (2017). Scaffold-free tissue-engineered allogenic adipose-derived stem cells promote meniscus healing. *Arthroscopy*.

[47] Qi Y., Yang Z., Ding Q., Zhao T., Huang Z., Feng G. (2016). Targeted transplantation of iron oxide-labeled, adipose-derived mesenchymal stem cells in promoting meniscus regeneration following a rabbit massive meniscal defect. *Experimental and Therapeutic Medicine*.

[48] Hatsushika D., Muneta T., Nakamura T. (2014). Repetitive allogeneic intraarticular injections of synovial mesenchymal stem cells promote meniscus regeneration in a porcine massive meniscus defect model. *Osteoarthritis and Cartilage*.

[49] Nakagawa Y., Muneta T., Kondo S. (2015). Synovial mesenchymal stem cells promote healing after meniscal repair in microminipigs. *Osteoarthritis and Cartilage*.

[50] Ozeki N., Muneta T., Matsuta S. (2015). Synovial mesenchymal stem cells promote meniscus regeneration augmented by an autologous Achilles tendon graft in a rat partial meniscus defect model. *Stem Cells*.

[51] Oda S., Otsuki S., Kurokawa Y., Hoshiyama Y., Nakajima M., Neo M. (2015). A new method for meniscus repair using type I collagen scaffold and infrapatellar fat pad. *Journal of Biomaterials Applications*.

[52] Koh R. H., Jin Y., Kang B. J., Hwang N. S. (2017). Chondrogenically primed tonsil-derived mesenchymal stem cells encapsulated in riboflavin-induced photocrosslinking collagen-hyaluronic acid hydrogel for meniscus tissue repairs. *Acta Biomaterialia*.

[53] Higashioka M. M., Chen J. A., Hu J. C., Athanasiou K. A. (2014). Building an anisotropic meniscus with zonal variations. *Tissue Engineering Part A*.

[54] McCorry M. C., Puetzer J. L., Bonassar L. J. (2016). Characterization of mesenchymal stem cells and fibrochondrocytes in three-dimensional co-culture: analysis of cell shape, matrix production, and mechanical performance. *Stem Cell Research & Therapy*.

[55] Niu W., Guo W., Han S., Zhu Y., Liu S., Guo Q. (2016). Cell-based strategies for meniscus tissue engineering. *Stem Cells International*.

[56] Bulgheroni E., Grassi A., Campagnolo M., Bulgheroni P., Mudhigere A., Gobbi A. (2016). Comparative study of collagen versus synthetic-based meniscal scaffolds in treating meniscal deficiency in young active population. *Cartilage*.

[57] Stone K. R., Rodkey W. G., Webber R., McKinney L., Steadman J. R. (1992). Meniscal regeneration with copolymeric collagen scaffolds. In vitro and in vivo studies evaluated clinically, histologically, and biochemically. *The American Journal of Sports Medicine*.

[58] Zaffagnini S., Grassi A., Marcheggiani Muccioli G. M. (2015). Two-year clinical results of lateral collagen meniscus implant: a multicenter study. *Arthroscopy*.

[59] Leroy A., Beaufils P., Faivre B., Steltzlen C., Boisrenoult P., Pujol N. (2017). Actifit® polyurethane meniscal scaffold: MRI and functional outcomes after a minimum follow-up of 5 years. *Orthopaedics & Traumatology, Surgery & Research*.

[60] Baynat C., Andro C., Vincent J. P. (2014). Actifit synthetic meniscal substitute: experience with 18 patients in Brest, France. *Orthopaedics & Traumatology, Surgery & Research*.

[61] Yuan Z., Liu S., Hao C. (2016). AMECM/DCB scaffold prompts successful total meniscus reconstruction in a rabbit total meniscectomy model. *Biomaterials*.

[62] Merriam A. R., Patel J. M., Culp B. M., Gatt C. J., Dunn M. G. (2015). Successful total meniscus reconstruction using a novel fiber-reinforced scaffold: a 16- and 32-week study in an ovine model. *The American Journal of Sports Medicine*.

[63] Patel J. M., Merriam A. R., Culp B. M., Gatt C. J., Dunn M. G. (2016). One-year outcomes of total meniscus reconstruction using a novel fiber-reinforced scaffold in an ovine model. *The American Journal of Sports Medicine*.

[64] Lee C. H., Rodeo S. A., Fortier L. A., Lu C., Erisken C., Mao J. J. (2014). Protein-releasing polymeric scaffolds induce fibrochondrocytic differentiation of endogenous cells for knee meniscus regeneration in sheep. *Science Translational Medicine*.

[65] Pan Z., Wu Y., Zhang X. (2017). Delivery of epidermal growth factor receptor inhibitor via a customized collagen scaffold promotes meniscal defect regeneration in a rabbit model. *Acta Biomaterialia*.

[66] Inchingolo F., Tatullo M., Marrelli M. (2010). Trial with platelet-rich fibrin and Bio-Oss used as grafting materials in the treatment of the severe maxillar bone atrophy: clinical and radiological evaluations. *European Review for Medical and Pharmacological Sciences*.

[67] Choukroun J., Diss A., Simonpieri A. (2006). Platelet-rich fibrin (PRF): a second-generation platelet concentrate. Part IV: clinical effects on tissue healing. *Oral Surgery, Oral Medicine, Oral Pathology, Oral Radiology, and Endodontics*.

[68] Dohan Ehrenfest D. M., Andia I., Zumstein M. A., Zhang C. Q., Pinto N. R., Bielecki T. (2014). Classification of platelet concentrates (platelet-rich plasma-PRP, platelet-rich fibrin-PRF) for topical and infiltrative use in orthopedic and sports medicine: current consensus, clinical implications and perspectives. *Muscles, Ligaments and Tendons Journal*.

[69] Wong C. C., Kuo T. F., Yang T. L. (2017). Platelet-rich fibrin facilitates rabbit meniscal repair by promoting meniscocytes proliferation, migration, and extracellular matrix synthesis. *International Journal of Molecular Sciences*.

[70] Mordecai S. C., Al-Hadithy N., Ware H. E., Gupte C. M. (2014). Treatment of meniscal tears: an evidence based approach. *World Journal of Orthopedics*.

[71] Osawa A., Harner C. D., Gharaibeh B. (2013). The use of blood vessel-derived stem cells for meniscal regeneration and repair. *Medicine and Science in Sports and Exercise*.

[72] Seol D., Zhou C., Brouillette M. J. (2017). Characteristics of meniscus progenitor cells migrated from injured meniscus. *Journal of Orthopaedic Research*.

[73] Kurth T. B., Dell'accio F., Crouch V., Augello A., Sharpe P. T., De Bari C. (2011). Functional mesenchymal stem cell niches in adult mouse knee joint synovium in vivo. *Arthritis and Rheumatism*.

[74] Decker R. S., Um H. B., Dyment N. A. (2017). Cell origin, volume and arrangement are drivers of articular cartilage formation, morphogenesis and response to injury in mouse limbs. *Developmental Biology*.

[75] Bhattacharjee M., Coburn J., Centola M. (2015). Tissue engineering strategies to study cartilage development, degeneration and regeneration. *Advanced Drug Delivery Reviews*.

[76] Occhetta P., Studle C., Barbero A., Martin I. (2016). Learn, simplify and implement: developmental re-engineering strategies for cartilage repair. *Swiss Medical Weekly*.

[77] Roelofs A. J., Zupan J., Riemen A. H. K. (2017). Joint morphogenetic cells in the adult mammalian synovium. *Nature Communications*.

[78] Mak J., Jablonski C. L., Leonard C. A. (2016). Intra-articular injection of synovial mesenchymal stem cells improves cartilage repair in a mouse injury model. *Scientific Reports*.

[79] Qu F., Holloway J. L., Esterhai J. L., Burdick J. A., Mauck R. L. (2017). Programmed biomolecule delivery to enable and direct cell migration for connective tissue repair. *Nature Communications*.

[80] Bhargava M. M., Hidaka C., Hannafin J. A., Doty S., Warren R. F. (2005). Effects of hepatocyte growth factor and platelet-derived growth factor on the repair of meniscal defects in vitro. *In Vitro Cellular & Developmental Biology - Animal*.

[81] Shen W., Chen J., Zhu T. (2014). Intra-articular injection of human meniscus stem/progenitor cells promotes meniscus regeneration and ameliorates osteoarthritis through stromal cell-derived factor-1/CXCR4-mediated homing. *Stem Cells Translational Medicine*.

[82] Yuan X., Eng G. M., Arkonac D. E., Chao P. H. G., Vunjak-Novakovic G. (2015). Endothelial cells enhance the migration of bovine meniscus cells. *Arthritis & Rheumatology*.

[83] Narita A., Takahara M., Sato D. (2012). Biodegradable gelatin hydrogels incorporating fibroblast growth factor 2 promote healing of horizontal tears in rabbit meniscus. *Arthroscopy*.

[84] Ozeki N., Muneta T., Koga H. (2013). Transplantation of Achilles tendon treated with bone morphogenetic protein 7 promotes meniscus regeneration in a rat model of massive meniscal defect. *Arthritis and Rheumatism*.

[85] Forriol F., Ripalda P., Duart J., Esparza R., Gortazar A. R. (2014). Meniscal repair possibilities using bone morphogenetic protein-7. *Injury*.

[86] Zhang S., Matsushita T., Kuroda R. (2016). Local administration of simvastatin stimulates healing of an avascular meniscus in a rabbit model of a meniscal defect. *The American Journal of Sports Medicine*.

[87] McNulty A. L., Moutos F. T., Weinberg J. B., Guilak F. (2007). Enhanced integrative repair of the porcine meniscus in vitro by inhibition of interleukin-1 or tumor necrosis factor *α*. *Arthritis & Rheumatism*.

[88] Freymann U., Metzlaff S., Krüger J. P. (2016). Effect of human serum and 2 different types of platelet concentrates on human meniscus cell migration, proliferation, and matrix formation. *Arthroscopy*.

[89] Dhurat R., Sukesh M. (2014). Principles and methods of preparation of platelet-rich plasma: a review and author’s perspective. *Journal of Cutaneous and Aesthetic Surgery*.

[90] Sanchez-Gonzalez D. J., Mendez-Bolaina E., Trejo-Bahena N. I. (2012). Platelet-rich plasma peptides: key for regeneration. *International Journal of Peptides*.

[91] Freymann U., Degrassi L., Kruger J. P., Metzlaff S., Endres M., Petersen W. (2017). Effect of serum and platelet-rich plasma on human early or advanced degenerative meniscus cells. *Connective Tissue Research*.

[92] Robinson K. R., Messerli M. A. (2003). Left/right, up/down: the role of endogenous electrical fields as directional signals in development, repair and invasion. *BioEssays*.

[93] Baker B., Becker R. O., Spadaro J. (1974). A study of electrochemical enhancement of articular cartilage repair. *Clinical Orthopaedics and Related Research*.

[94] Baker B., Spadaro J., Marino A., Becker R. O. (1974). Electrical stimulation of articular cartilage regeneration. *Annals of the New York Academy of Sciences*.

[95] Yuan X., Arkonac D. E., Chao P. H., Vunjak-Novakovic G. (2014). Electrical stimulation enhances cell migration and integrative repair in the meniscus. *Scientific Reports*.

[96] Reing J. E., Zhang L., Myers-Irvin J. (2009). Degradation products of extracellular matrix affect cell migration and proliferation. *Tissue Engineering Part A*.

